# Acute Health Impacts of the Southeast Asian Transboundary Haze Problem—A Review

**DOI:** 10.3390/ijerph16183286

**Published:** 2019-09-06

**Authors:** Kang Hao Cheong, Nicholas Jinghao Ngiam, Geoffrey G. Morgan, Pin Pin Pek, Benjamin Yong-Qiang Tan, Joel Weijia Lai, Jin Ming Koh, Marcus Eng Hock Ong, Andrew Fu Wah Ho

**Affiliations:** 1 Science and Math Cluster, Singapore University of Technology and Design, Singapore 487372, Singapore; 2 Division of Neurology, Department of Medicine, National University Health System, Singapore 119074, Singapore; 3 School of Public Health, The University of Sydney, Sydney, NSW 2006, Australia; 4 Department of Emergency Medicine, Singapore General Hospital, Singapore 169608, Singapore; 5 Health Services & Systems Research, Duke-NUS Medical School, Singapore 169857, Singapore; 6 SingHealth Duke-NUS Emergency Medicine Academic Clinical Programme, Singapore 169857, Singapore; 7 National Heart Research Institute Singapore, National Heart Centre, Singapore 169609, Singapore; 8 Cardiovascular & Metabolic Disorders Programme, Duke-NUS Medical School, Singapore 169857, Singapore

**Keywords:** Big data, data analytics, transboundary, haze, air pollution, fire, healthcare, environmental epidemiology, public health

## Abstract

Air pollution has emerged as one of the world’s largest environmental health threats, with various studies demonstrating associations between exposure to air pollution and respiratory and cardiovascular diseases. Regional air quality in Southeast Asia has been seasonally affected by the transboundary haze problem, which has often been the result of forest fires from “slash-and-burn” farming methods. In light of growing public health concerns, recent studies have begun to examine the health effects of this seasonal haze problem in Southeast Asia. This review paper aims to synthesize current research efforts on the impact of the Southeast Asian transboundary haze on acute aspects of public health. Existing studies conducted in countries affected by transboundary haze indicate consistent links between haze exposure and acute psychological, respiratory, cardiovascular, and neurological morbidity and mortality. Future prospective and longitudinal studies are warranted to quantify the long-term health effects of recurrent, but intermittent, exposure to high levels of seasonal haze. The mechanism, toxicology and pathophysiology by which these toxic particles contribute to disease and mortality should be further investigated. Epidemiological studies on the disease burden and socioeconomic cost of haze exposure would also be useful to guide policy-making and international strategy in minimizing the impact of seasonal haze in Southeast Asia.

## 1. Introduction

Air pollution has emerged as one of the largest global environmental health threats in modern times [1]. Across countries, the focus of environmental disease burden has largely shifted from communicable diseases to noncommunicable diseases in adults [2], as medical technology and intervention capabilities continue to progress. This trend points towards an urgent necessity in investigating environment-associated noncommunicable diseases and deriving suitable mitigation measures, both in consideration of public health and the socioeconomic security of communities and nation states. Indeed, a large amount of research has demonstrated the deleterious health effects of exposure to air pollution in respiratory and cardiovascular illnesses, with the young and the elderly being the most vulnerable [3].

Similarly, air pollution has been an issue of paramount importance in Southeast Asia with Southeast Asian and Western Pacific regions bearing most of the burden [4]. Regional air quality in Southeast Asia has been seasonally affected by the transboundary haze problem in past decades, which has typically been the result of vegetation fires from “slash-and-burn” farming methods common in the area. These low-cost methods involve the burning of trees and plants in the farming fields to create a nutrient-rich ash layer largely devoid of weeds on which crops can be grown. This process releases large amounts of chemical and particulate airborne pollution. This has had immense economic, political, and health-related implications [5,6], and the near-catastrophic extent of the seasonal haze episodes has prompted concerted efforts to mitigate the situation. Most recently, the Association of Southeast Asian Nations (ASEAN) Agreement on Transboundary Haze Pollution has been ratified by all countries within ASEAN, pledging support to reduce haze pollution in Southeast Asia [7].

The regional nature of the problem has been the result of transboundary winds that transport the haze throughout more than half of the ASEAN countries, including Singapore, Indonesia, Malaysia, Brunei, Thailand, and the Philippines [8,9,10]. In addition, specific weather and climate conditions unique to the area, such as the dry weather conditions consequent of the El Nino-Southern Oscillation and positive Indian Ocean Dipole, have further worsened the severity of the haze engulfing the affected countries [11,12].

In general, the seasonal transboundary haze arises from combustion-associated smoke emissions, which comprise high concentrations of particulate matter fine enough to be carried airborne. A vast majority of the particles are less than 2.5 microns in size (PM2.5), and can therefore easily be suspended in wind currents for long periods of time, and are also sufficiently small to penetrate deep into the human respiratory tract [13,14]. In an acute setting, these seasonal haze episodes contribute to worsening asthma problems and other respiratory-related symptoms, but the long-term health risks of intermittent extreme episodes of seasonal haze exposure remains largely unclear. Some studies have suggested an increased risk of chronic diseases such as lung cancers, but these have been in the context of chronic exposure [15,16]. It has not been established if seasonal short-term, high-level repeated haze exposures confer similar increased cancer risks and other long-term health outcomes, as had been observed in North American or European urban settings that do not experience these extreme seasonal fluctuations in air pollution severity. A recent review by Reid et al. has also indicated a consistent demonstration of increased short-term respiratory morbidity and mortality consequent of seasonal smoke exposure from episodic wildfires [17], suggesting appropriate pertinence in comprehensively exploring the impact of the seasonal haze on acute health risks in Southeast Asia.

We recognize that the transboundary haze problem forms a growing public health concern. A multitude of recent studies have begun to examine the health effects of the Southeast Asian seasonal haze, in isolation and with regard to specific organ systems. The results of these research efforts help quantify the severity of the health burden of the annually recurring haze episodes, which in turn aids the formulation of regional and country-specific institutional policy responses. The purpose of this review paper is to close an important gap in the literature by synthesizing existing knowledge on the public health impact of the Southeast Asian transboundary haze. With a better understanding and a more extensive knowledge base, governments and organizations may be able to enact more effective response and resolution strategies in the near future.

## 2. Findings and Results

Our study involved a search of the following terms in PubMed, Google Scholar, and Cochrane Library: ‘haze’, ‘transboundary haze’, ‘seasonal haze’, ‘South-east Asia’, ‘Asia’, ‘Pollutant Standards Index’, ‘air pollution’, ‘respiratory diseases’, ‘cardiovascular diseases’, ‘neurological diseases’, ‘psychological stress’, ‘psychosomatic symptoms’, ‘physical symptoms’, and ‘mortality’. We focused mainly on studies within the context of Southeast Asia; and some studies pertaining to Asia and elsewhere were used as reference point as and when required. We identified 44 articles for a full-text review after accounting for the duplicates and irrelevant articles, of which 31 directly address types of diseases and findings from haze-related health issues.

**Figure 1:**
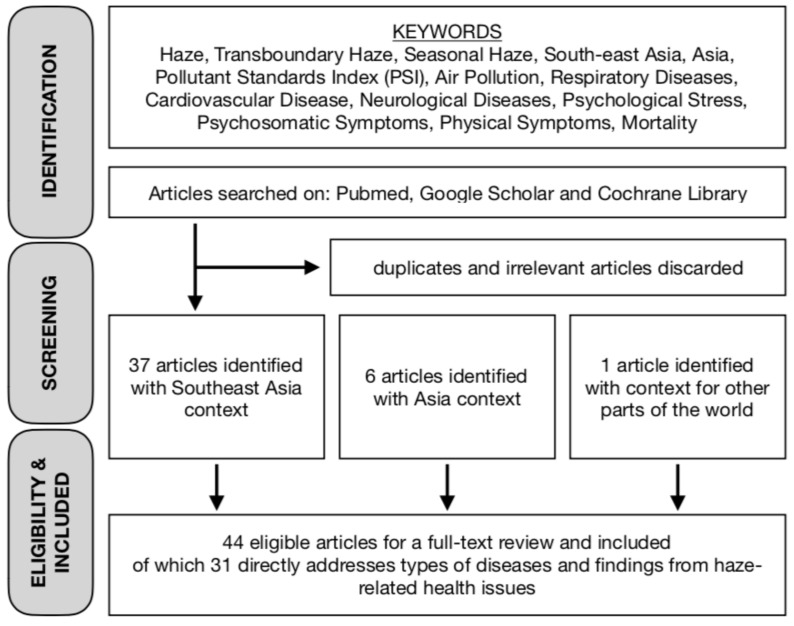
Flowchart of paper selection.

**Figure 2:**
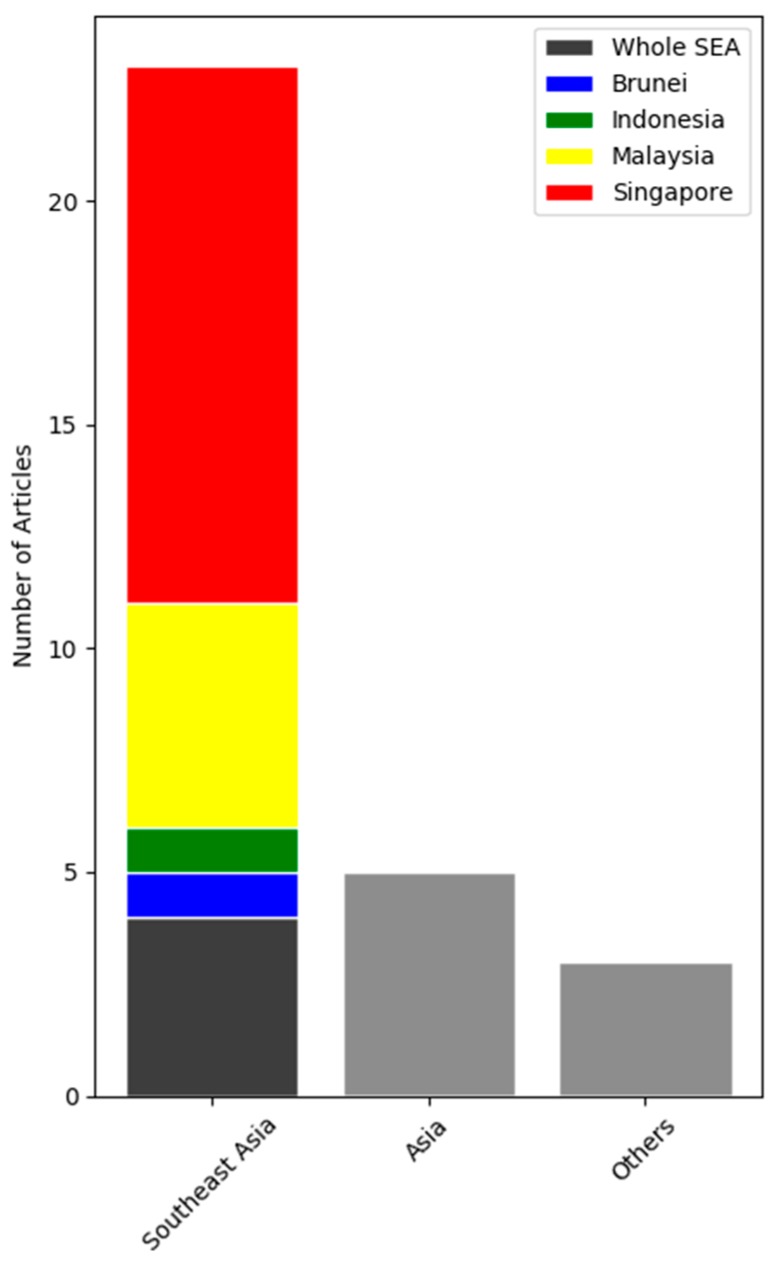
Number of articles relating to haze and health studies by region and country.

In the subsequent sections, we will give an overview of the health implications of the transboundary haze problem based on the results obtained from these studies. Figure 1 illustrates the decision process in our choice of papers undertaken in this review. Figure 2 and Table 1 provide a break-down of the selected papers.

### 2.1. Physical Symptoms and Psychological Impact

Small-scale retrospective studies have consistently demonstrated that a number of physical symptoms are exacerbated or worsened by the seasonal haze. These symptoms included sore throat or dry mouth, nose discomfort, eye discomfort, headache, shortness of breath, as well as skin irritation [18,19]. It was not always clear if these symptoms were exacerbations of underlying existing medical conditions (e.g., flare of endogenous eczema), or if the presence of new de novo symptoms brought on by the haze episodes. 

A study from Singapore has also shown that the seasonal haze is associated with mild psychological stress [20]. This was examined by means of a cross-sectional, self-administered questionnaire. The total IES-R (Impacts of Events Scale–Revised) score in this study of 298 subjects was 18.47 (±11.69), which indicated that the study population experienced mild psychological stress, not amounting to acute stress reaction [20].

These psychosomatic symptoms experiences may have been in part mechanistically explained by alterations in cerebral hemodynamics as a result of exposure to haze. In a prospective study on healthy subjects with transcranial Doppler investigations conducted prior and after haze exposure, it had been found that there was a modest but significant decrease in pulsatility index (PI) and resistivity index (RI) in the left middle cerebral artery after haze exposure. Haze exposure resulted in significantly lower mean PI and RI in symptomatic subjects compared to baseline parameters, but was not significantly different in the asymptomatic subjects [21].

It would appear overall that there is concordance from prior studies demonstrating the effect of haze on the presence of psychological symptoms, which may have been mechanistically explained by acute changes in cerebral hemodynamics after haze exposure. A summary is found in Table 1.

### 2.2. Respiratory Disease

Respiratory symptoms and complaints predominate during a haze episode, although they tend to be mild. In Singapore, during the 1997 haze episode, an increase in PM10 levels from 50 microg/m^3^ to 150 microg/m^3^ was accompanied by a 12% increase in cases of upper respiratory tract illness, and 26% increase in cases of rhinitis [22]. Such symptoms are commonly presented to primary care providers, but it was more common to see cases of more serious lower respiratory conditions such as bronchitis and emphysema in periods of haze exposure [22]. It was also established that the persons at greater risk of these conditions are the very young and the elderly, as well as those living in urban environments and working outdoors [23]. Some studies have established a “dose-dependent” relationship between haze exposure and respiratory symptoms, where higher Pollutant Standards Index (PSI) values are associated with more frequent respiratory symptoms [9].

Notably, patients with preexisting respiratory conditions may be at risk of significant worsening in periods of seasonal haze. There have been increases in hospital admissions for exacerbations of chronic obstructive pulmonary disease during haze episodes, with elderly patients above 65 years of age being the most vulnerable [24]. Hospitalizations for asthma have also been shown to increase by as much as 20% in Singapore [25]. This may be attributed to the deterioration of pulmonary function as a direct effect of airborne particulate matter in the transboundary haze. A study on children have also indicated significant reduction in pulmonary function during haze episodes in comparison to pre-exposure conditions, especially in girls; when pulmonary function testing was repeated again after the haze episode, there was only partial recovery as residual effects from haze exposure remained [26]. This suggests possible long-term health implications from persistent residual effects, or at least a significant recovery period associated with each haze episode.

In general, haze exposure has consistently shown association with respiratory symptoms. Studies on respiratory symptoms have also been helpful in describing a dose-dependent relationship between the severity of haze and the presence of respiratory symptoms.

### 2.3. Neurological Diseases

It has been established that long-term exposure to air pollution is associated with an increased risk of cerebrovascular disease [27]. Seasonal air quality deterioration in China, especially during cold winter months, when the pollution levels are 50% higher than baseline, has been previously associated with increased hospital admissions for stroke [28]. Beyond cerebrovascular diseases, other neurological conditions such as headaches and migraines are also more common after exposure to air pollution [28,29,30]. In Taiwan, studies have demonstrated that PM2.5 exposure in particular was independently associated with increased outpatient visits for headaches and migraines [31,32]. More recently, these trends have been demonstrated in Southeast Asia. A study from Singapore has demonstrated an association between haze exposure and an increased risk of stroke. Moderate and unhealthy levels of PSI correlated positively with stroke occurrence, with incidence risk ratios 1.10 (95% confidence interval 1.06 to 1.13) and 1.14 (95% confidence interval 1.03 to 1.25), respectively. This risk remained elevated 5 days after exposure [33].

The underlying pathophysiological mechanisms for haze exposure and neurological disease remain to be concretely elucidated. However, it has been postulated that some airborne particles in the transboundary haze may have vasoactive properties when inhaled, and may lead to alterations in cerebral hemodynamics [21]. It appeared that some subjects were more susceptible to these changes than others, and that alterations in the cerebral hemodynamics were correlated with the observed neurological symptoms, such as headaches. However, it is as yet unclear if the changes in cerebral hemodynamics can lead to an increased risk of stroke, or whether patients with existing cerebrovascular disease were more susceptible compared to healthy subjects.

Beyond respiratory diseases, haze exposure has also been associated with diseases of other organ systems. Although pathophysiological mechanisms and causality have not been robustly demonstrated, previous studies have consistently demonstrated the association of haze exposure with cerebrovascular disease and migraine. 

### 2.4. Cardiovascular Diseases

Studies have postulated that mechanisms for cardiovascular disease after exposure to air pollution may be related to the direct toxicity of the inhaled particulate matter to the cardiovascular system or by indirect injury causing systemic inflammation, oxidative stress, and accelerated atherosclerosis [34,35]. An association between seasonal haze exposure and the occurrence of acute myocardial infarction has also been suggested by studies in Singapore [36,37]. It was found that the PSI was significantly associated with increased acute myocardial infarction occurrence, with each 30-unit increment in PSI being associated with IRR of 1.04 (95% CI 1.03–1.06), as reflected in Table 2 [37].

Similar to neurological disease, previous authors have suggested pathophysiological mechanisms for the association between haze exposure and cardiovascular disease. However, although the mechanisms remain unclear, the association has been shown consistently.

### 2.5. Mortality

Out-of-hospital cardiac arrests have been shown to be more frequent during periods of seasonal haze exposure, when haze severity reach moderate and unhealthy PSI levels. In a 12 year time series analysis, it was demonstrated that a 10 μg/m^3^ increase in particulate matter was associated with significant increases in nonaccidental (PM10 ER: 0.627%; 95% confidence interval (CI): 0.260–0.995% and PM2.5 ER: 0.660%; 95% CI: 0.204–1.118%) and cardiovascular deaths (PM10 ER: 0.897; 95% CI: 0.283–1.516 and PM2.5 ER: 0.883%; 95% CI: 0.121–1.621%) [38]. This study found that the impact of nonparticulate matter pollutants was minimal. Other studies on air pollution in other regions have similarly demonstrated increased cardiovascular mortality associated with higher levels of air pollution, with particular emphasis on PM2.5 exposure [39]. Similar trends had been observed in the seasonal haze episodes in Malaysia from 2000 to 2007, where higher mortality was observed with incrementally higher levels of PM10 exposure. 

There was an observable lag or latency effect, in which the highest mortality was seen between 2 and 5 days after the exposure to haze [40]. The population at greatest risk was, again, the elderly, above 60 years of age, and children, under 14 years of age. In the elderly, the risk of cardiovascular death was up to twice as high after exposure to severe haze episodes [6].

Overall mortality has also been consistently shown to be high during periods of haze exposure. Of note, the extremes of age (elderly and young) have been the most vulnerable to this effect.

## 3. Discussion

The vast majority of studies investigating the acute health impact of seasonal haze in Southeast Asia has demonstrated an association between haze exposure and deleterious health effects, which range from physical and psychosomatic symptoms to respiratory, neurological, cardiovascular disease and mortality [18,20,21,23,24,25,26,27,28,29,30,31,32,33,34,35,41,42]. However, the evidence as a whole remains largely inconclusive for several reasons. 

The first limitation of these studies is the availability of data. The ASEAN consist of many nations that currently lack robust air quality monitoring infrastructure to enable reliable data collection. The location of health events may be geographically distant from the nearest air pollution monitoring site. This may be partially dealt with using extrapolative modeling or satellite image plots but these have not been utilized in studies originating from this region. At the same time, in certain nations there is additionally the reluctance of government agencies to release pollutant data for research. 

Secondly, some of the larger studies from the region suffer from using an air quality index as the exposure of interest. For example, in Singapore, PSI has been shown to correlate with acute myocardial infarction, out-of-hospital cardiac arrest, and acute ischemic stroke [33,37,38]. While PSI is useful as a public communication tool due to its ability to summarize the state of pollution into a single number which the public can easily interpret, in comparative research it becomes ineffective. Also, the study of threshold effects is compromised due to its nonlinear scale. 

Thirdly, the studies which are predominantly of time series or case crossover designs, suffer from fundamental limitations of ecological studies. In addition, the small size and retrospective nature of most studies limit their usefulness. These designs also make it difficult to isolate the effect of transboundary haze from endogenous sources of pollutants such as industrial and vehicular sources. A future direction would be to study some of the high-quality cohort studies currently housed within the region. 

Lastly, a major limitation has been the difficulty in quantifying the true exposure of individuals to the transboundary haze. Furthermore, differences in socioeconomic status and working exposure (i.e., outdoor work versus indoor air-conditioned environment) may also influence the degree of true exposure to the environmental haze. The subjective health beliefs of individuals, in the form of the perceived threat that the haze poses, may also have influenced the duration of time spent outdoors, or the enactment of protective measures, such as the usage of masks or air filters. These differences are difficult to quantify, and most studies did not fully explore if individuals with differing exposure levels present different health implications.

Most of the data on seasonal haze originates from the countries of Singapore, Malaysia, and Brunei. There is a paucity of data from the most affected countries such as Indonesia, Thailand and Philippines. Data from these severely affected countries would be valuable in adding to the comprehensive understanding of the epidemiology and disease associations in the region.

Reassuringly, many studies on health outcomes had spanned a number of years. The trends of increased stroke and cardiovascular mortality had not just been demonstrated in an isolated haze season, but consistently demonstrated yearly with each seasonal haze exposure [28,29,31]. The studies had also consistently demonstrated that young children and the elderly were at the highest risk of health effects after exposure to haze [24,25], which may be related to a poorer or impaired immune system, as well as the potential presence of preexisting health conditions that may have been easily exacerbated by the environmental haze exposure.

We also note that there has been a shift in research effort towards examining the mechanism, toxicology and pathophysiology in which exposure to haze contributes to disease. A study from Singapore has demonstrated changes in cerebral hemodynamics after haze exposure and its association with the presence of psychosomatic symptoms [21]. However, the subjects studied were all young and healthy volunteers, and it is unclear if differential effects were present in those with preexisting cerebrovascular disease. Longitudinal studies were also lacking to evaluate if long-term health outcomes were affected by these differences in cerebral hemodynamics.

Studies on a cellular level have shown that human epithelial cell lines had decreased cell viability and increased cell death when exposed to PM2.5 samples collected during a severe haze episode. There were low levels of glutathione and caspase-3/7 in the cells affected, which may have led to higher levels of reactive oxygen species that cause oxidative stress and eventual cell death. These effects were attributed to metal-bound particulate matter and polycyclic aromatic hydrocarbons in the haze, and, given this mechanism, may contribute to inflammation in the airways and lungs, and consequently respiratory symptoms with recurrent haze exposure [13]. More studies evaluating the measurement of particulate matter in humans such as from blood or urine samples, or via other biomarkers may be useful to improve further understanding and quantify the toxic effects of haze exposure.

While greater awareness of seasonal haze and its health effects has led to growing academic interest, there still remains a scarcity of data on the effects of seasonal haze and its burden on primary care providers. Minor ailments that require attention may be seen in various contexts by general practitioners, or in some cases, emergency department visits. Collating data on the increase in volume of cases experienced by primary care providers or emergency departments during haze exposure would help to guide important health policies.

## 4. Conclusions and Outlook

The seasonal transboundary haze problem in Southeast Asia has become recurrent and is of significant public health concern. Although there has been a paucity of data from several low–middle-income countries, existing studies arising from some of the affected countries suggest consistent deleterious effects on psychological, respiratory cardiovascular, neurological morbidity, and mortality; and this motivates future work.

Addressing the limitations of the current literature in this area, research in this field may be enhanced in several ways. First is to improve the quality of the exposure data. This may mean government investment into developing air quality monitoring infrastructure. In other countries which already possess this, it then becomes necessary to navigate the bureaucratic barriers to data availability.

Secondly, future studies should move beyond describing disease association, to quantify impact on health service utilization as this allows another dimension of the quantification of the economic cost of transboundary haze episodes and also facilitates in the planning of healthcare resources. These would involve events such as ambulance callouts, emergency department visits, and hospital admissions. Having such data will then allow the evaluation of public health interventions such as mask distribution exercises or school closure policies.

Future prospective and longitudinal studies are warranted to quantify the long-term health effects of recurrent but intermittent exposure to high levels of seasonal haze. These studies should look into the mechanism, toxicology and pathophysiology by which these toxic particles contribute to disease and mortality. In-depth study of retroactive measures to provide healthcare to those afflicted by haze related illnesses, as well as to study the severity of health effects due to seasonal haze, can prepare the healthcare sector and authorities in anticipation for seasonal haze. While taking retrospective action may decrease the number of those who suffer from long-term health affliction, being active in predicting seasonal haze and its severity should be at the forefront so that action can be taken as a preventive measure.

With the continued progress of technology, it is also hoped that artificial intelligence-based approaches can be used to forecast PM2.5 during haze episodes [43,44]. In India, an advanced machine learning technique was used to effectively forecast haze episodes over urbanized areas. The approach employed the combined use of neural networks and fuzzy logic for predicting PM2.5 levels during haze conditions. These novel techniques were compared against conventional methods; it was convincingly shown that artificial intelligence techniques had significantly better agreement with the observed values, especially in the context of urbanized districts like Delhi [43]. 

We also emphasize that epidemiology studies on the disease burden and socioeconomic cost of haze exposure would be useful to guide policy and strategy in minimizing the health impact of the seasonal haze in Southeast Asia. However, preventive and retrospective action will not solve the issues or eliminate the source of the haze. Therefore, we conclude this review paper by encouraging farmers to adopt more sustainable practices with effective fire and forest management to “tackle the haze issue at its root.” The slash-and-burn agricultural practice may be economical for landowners, but it has a great socioeconomic burden on a larger scale. Whenever possible, related authorities should aid greatly in incentivizing the shift towards alternatives, especially when considering cost balance to be the primary impetus in the widespread use of slash-and-burn techniques in the first place. 

## Figures and Tables

**Table 1:** Key findings of haze-related health issues.

Article	Region/Study country/Period of Study	Key Findings
Chew, et al. (1995) [25]	SEA/Singapore/September to October 1994	An increase in emergency room attendances for acute childhood asthma in two large general hospitals in Singapore
Hashim, et al. (1998) [26]	SEA/Malaysia/July to November 1997	Significant reductions (mean 18%) in pulmonary function among children during and after the episode when compared to the pre-episode period. The mean reduction in percentage predicted FEV1 and FVC and ratio FEV1/FVC during the haze were lower among the girls (21, 19, and 8% respectively) than among the boys (16, 10, and 5% respectively).
Emmanuel(2000) [22]	SEA/Singapore/data from August to November 1997	Findings from the health impact of the haze showed that there was a 30% increase in outpatient attendance for haze-related conditions. An increase in PM_10_ levels from 50 μg/m^3^ to 150 μg/m^3^ was significantly associated with increases of 12% of upper respiratory tract illness, 19% asthma, and 26% rhinitis.
Tan, et al. (2000) [19]	SEA/Singapore/29 September to 27 October 1997 and 21 November to 5 December 1997	The study examined the association between acute air pollution caused by biomass burning and peripheral white blood cell counts in humans. Serial measurements of the WBC count made during the 1997 Southeast Asian Smoke-haze (Sep 29 to Oct 27) were compared with a period after the haze cleared (Nov 21 to Dec 5) using peripheral blood polymorphonuclear leukocytes (PMN) band cells to monitor marrow release. Atmospheric pollution caused by biomass burning is associated with elevated circulating band cell counts in humans because of the increased release of PMN precursors from the marrow—this response may contribute to the pathogenesis of cardiorespiratory morbidity associated with acute air pollution.
Odihi (2001) [9]	SEA/Brunei/September 1997 to September 1998	The deleterious effects of haze appeared skewed towards the young (age: 1–5 years) and the aged (≥60 years). A higher proportion of urban population was more adversely affected than in rural areas and, other things being equal, a higher proportion of outdoor workers were more adversely affected by haze than their indoor counterparts. Conjunctivitis related cases did not have any significant increase during the exposure period.
Sastry (2002) [6]	SEA/Malaysia/April to November 1997	Total mortality associated with a 100 μg/m^3^ increase in PM_10_ concentrations for Kuala Lumpur, associated relative risk is 1.07. For one segment of the Malaysian population—those aged 65 to 74 in Kuala Lumpur—there was an upward shift in mortality that lasted at least a few weeks.
Frankenberg, et al. (2005) [23]	SEA/Indonesia/1993 and 1997	Comparisons of the health of the population living in haze areas with the health of those in other areas substantially overestimated the “effect” of the fires because of time-varying location-specific unobserved heterogeneity in health status. One in 3 adult respondents over the age of thirty reported coughing as the major health issue during the 1997 haze episode.
Mott, et al. (2005) [24]	SEA/Malaysia (Kuching)/ January 1995 to December 1998	Comparisons of long-term cardiorespiratory disease classifications were done. Significant increases in respiratory hospitalizations, particularly those due to asthma, were observed in the 19–39- and 40–64-year-old categories. Persons over age 65 with prior hospitalizations for respiratory diseases were significantly more likely than others to be rehospitalized for their conditions during the forest fire.
Szyszkowicz, et al. (2009) [29]	Others/Canada (Edmonton)/1992 to 2002	Findings provide preliminary evidence of an association between air pollution and emergency department visits for migraine and nonspecific headache. Findings were most consistent for particulate matter.
Szyszkowicz, et al. (2009) [30]	Others/Canada (Edmonton, Hali- fax, Ottawa, Toronto, and Vancouver)/data from 11518 days for five cities.	For female ED visits for migraine, positive associations were observed during the warm season for sulfur dioxide (SO_2_), and in the cold season for particulate matter (PM_2.5_) exposures lagged by 2-days. The percentage increase in daily visits was 4.0% (95% CI: 0.8–7.3) for SO_2_ mean level change of 4.6 ppb, and 4.6% (95% CI: 1.2–8.1) for PM_2.5_ mean level change of 8.3 μg/m^3^. For male ED visits for headache, the largest association was obtained during the warm season for nitrogen dioxide (NO_2_), which was 13.5% (95% CI: 6.7–20.7) for same day exposure.
Yang, et al. (2012) [39]	Asia/China (Guangzhou)/2007 to 2008	The averaged PM_2.5_ concentration in 2007–2008 was 70.1 μg/m^3^ in Guangzhou, which was approximately seven times higher than the WHO Air Quality Guidelines. An increase of 10 μg/m^3^ in 2-day moving average (lag01) concentration of PM_2.5_ corresponds to 0.90% [95% confidence interval (CI): 0.55, 1.26%] increase of total mortality, 1.22% (95% CI: 0.63, 1.68%) increase of cardiovascular mortality, and 0.97% (95% CI: 0.16, 1.79%) increase of respiratory mortality. The associations were stronger in the elderly (aged 65 years or more), in females, and in those with low education level, but the differences were statistically insignificant.
Andersen, et al. (2012) [27]	Others/Denmark (Copenhagen and Aarhus)/data from 1971 to 2006	Over a mean follow-up of 9.8 years of 52 215 eligible subjects, there were 1984 (3.8%) first-ever (incident) hospital admissions for stroke of whom 142 (7.2%) died within 30 days. Detected borderline significant associations between mean nitrogen dioxide levels at residence since 1971 and incident stroke (hazard ratio, 1.05; 95% CI, 0.99–1.11, per interquartile range increase) and stroke hospitalization followed by death within 30 days (1.22; 1.00–1.50). The associations were strongest for nonspecified and ischemic strokes, whereas no association was detected with hemorrhagic stroke.
Abba, et al. (2012) [16]	Asia/India (Mumbai) /2007 to 2008	The average outdoor PM_2.5_ mass concentrations at control, kerb, residential and industrial sites were 69+21, 84+32, 89+34, 95+36 μg/m^3^. The sums of PAHs in PM_2.5_ at same above four sites were 35.27 + 2.10, 42.96 + 2.49, 175.76 + 8.95 and 90.78 + 4.74 μg/m^3^, respectively. Estimating the carcinogenic potential of PAHs with equivalents of Benzo(a)pyrene (BaPE). The maximum value of BaPE (18.8) was reported in the residential site.
Xiang, et al. (2013) [28]	Asia/China (Wuhan)/2006 to 2008	Time stratified case crossover design by season (April–September and October–March) was performed to assess effects of pollutant on stroke hospital admissions. Exposure to NO_2_ is significantly associated with stroke hospitalizations during the cold season in Wuhan, China when pollution levels are 50% greater than in the warm season.
Marlier, et al. (2013) [8]	SEA/All/1997 to 2006	During strong El Niño years, fires contribute up to 200 μg/m^3^ and 50 ppb in annual average fine particulate matter (PM_2.5_) and ozone (O_3_) surface concentrations near fire sources, respectively. Corresponds to a fire contribution of 200 additional days per year that exceed the World Health Organization (WHO) 50 μg/m^3^ 24-hour PM_2.5_ interim target (IT-2) and an estimated 10,800 (6800–14,300) person (~2%) annual increase in regional adult cardiovascular mortality.
Pavagadhi, et al. (2013) [13]	SEA/Singapore/21 to 29 October 2010	Physicochemical and toxicological characteristics of both haze and non-haze aerosols were evaluated. The average mass concentration of PM_2.5_ (PM with aerodynamic diameter of ≤2.5 μm) increased by a factor of 4 during the smoke haze period (107.2 μg/m^3^) as compared to that during the non-smoke haze period (27.0 μg/m^3^). Metal concentrations were also found to be higher in haze aerosols. Additionally, the percentage of metabolically active cells decreased significantly following a direct exposure to PM samples collected during the haze period.
Betha, et al. (2014) [5]	Southeast Asia (SEA)/All/June 2013	PM_2.5_ concentrations were elevated (up to 329 μg/m^3^) during the haze episode, compared to those during the non-haze period (11–21 μg/m^3^). There was a 10-fold increase in the concentration of K, an inorganic tracer of biomass burning. Health risk estimates revealed that the excessive lifetime carcinogenic risk to individuals exposed to biomass burning-impacted aerosols (18 ± 1 × 10^–6^) increased significantly (*p* < 0.05) compared to those who exposed to urban air (12 ± 2 × 10^–6^).
Sahani, et al. (2014) [40]	SEA/Malaysia (Klang Valley)/data from 2000 to 2007	A total of 88 haze days were identified in the Klang Valley region during the study period. A total of 126,822 cases of death were recorded for natural mortality where respiratory mortality represented 8.56% (*N* = 10,854). Haze events were found to be significantly associated with natural and respiratory mortality at various lags. For natural mortality, haze events at lagged 2 showed significant association with children less than 14 years old (Odd Ratio (OR) = 1.41; 95% Confidence Interval (CI) = 1.01–1.99). Respiratory mortality was significantly associated with haze events for all ages at lagged 0 (OR = 1.19; 95% CI = 1.02–1.40).
Yeo, et al. (2014) [18]	SEA/Singapore/25 June to 11 July 2013	Seventy-two consultations were conducted over the 3-week period, of which 26 (36.1%) were haze related, 18 (25%) were possibly haze related and 28 (38.9%) were non-haze related. The majority of haze-related complaints were respiratory, eye and skin- related.
Ho, et al. (2014) [20]	SEA/Singapore/21 June to 26 June 2013	Study was conducted between June 21 and June 26, 2013. Participants were recruited by online recruitment post. Participants were required to complete an online survey which was composed of demographics questionnaire, physical symptom checklist, perceived dangerous Pollutant Standard Index (PSI) value, and views on the N-95 mask and the Impact of Event Scale-Revised (IES-R). A total of 298 participants returned the completed study questionnaire. The respondents reported a mean number of 4.03 physical symptoms (S.D. = 2.6). The five most common physical symptoms include mouth or throat discomfort (68.8%), nose discomfort (64.1%), eye discomfort (60.7%), headache (50.3%), and breathing difficulty (40.3%). The total IES-R score was 18.47 (S.D. = 11.69) which indicated that the study population experienced mild psychological stress but not to the extent of acute stress reaction syndrome.
Reddington, et al. (2014) [10]	SEA/ Indonesia, Malaysia and Singapore/2004 to 2009	Fires in southern Sumatra account for the greatest percentage of the total fire enhancement to PM_2.5_ concentrations in Singapore (42–62%), with fires in central Sumatra and Kalimantan contributing 21–35% and 14–15%, respectively. Explored the impact of vegetation and peat fires on PM_2.5_ concentrations across other major cities in the region. Fires that contributed most to PM_2.5_ concentrations in Singapore also contributed substantially to the concentrations across the rest of the region. Jakarta, Palembang and Batam are mostly impacted by the fires in southern Sumatra (accounting for 51–74% of the total fire enhancement to PM_2.5_), whereas Kuala Lumpur and Pekanbaru are impacted most by fires in central Sumatra (accounting for 69–74% of the total fire enhancement to PM_2.5_). Targeting fire reduction efforts to improve air quality in Singapore will also improve air quality in other major cities in Indonesia and Malaysia.
Chen, et al. (2015) [31]	Asia/Taiwan (Taipei)/data from 2006 to 2011	No significant associations between PM_2.5_ levels and migraine visits were observed on cool days. On warm days, for the single pollutant model, there is a 13% increased clinic visits for migraine were significantly associated with PM_2.5_ levels.
Chang, et al. (2015) [32]	Asia/Taiwan (Taipei)/data from 2006 to 2011	Increased outpatient department (OPD) visits for headaches were significantly associated with levels of PM_2.5_ both on warm days (>23 °C) and cool days (<23 °C), with an interquartile range rise associated with a 12% (95% CI = 10–14%) and 3% (95% CI = 1–5%) elevation in OPD visits for headaches, respectively.
Koplitz, et al. (2016) [12]	SEA/ Indonesia, Malaysia and Singapore/September to October 2015 compared to haze of September to October 2006	Using the adjoint of the GEOS-Chem chemical transport model, they calculated the influence of potential fire emissions across the domain on smoke concentrations in three receptor areas – Indonesia, Malaysia and Singapore during the haze episode of 2006. The model framework introduced in this study identified areas where land use management to reduce and/or avoid fires would yield the greatest benefit to human health, both nationally and regionally.
Khan, et al. (2016) [15]	SEA/Malaysia/July to September 2013 and January to February 2014	Samples are collected from a building 65 m above sea level, 1 km from the main highway road. The hazard quotient for four selected metals (Pb, As, Cd, and Ni) in PM_2.5_ mass was highest in PM_2.5_ mass from the coal burning source and least in PM_2.5_ mass originating from the mineral/road dust source. The main carcinogenic heavy metal of concern to health at the location was As. Overall, the associated lifetime cancer risk posed by the exposure of hazardous metals in PM_2.5_ is 3–4 per 1,000,000 people at the location.
Ho, et al. (2018) [33]	SEA/Singapore/data from 2010 to 2015	There were 29,384 ischemic stroke cases. Moderate and unhealthy Pollutant Standards Index levels showed association with stroke occurrence, with incidence risk ratio 1.10 (95% confidence interval 1.06 to 1.13) and 1.14 (95% confidence interval 1.03 to 1.25), respectively. The association was significant in subgroups aged 65 years or older, women, Chinese, nonsmokers and those with history of diabetes, hypertension, and hyperlipidemia.
Yap, et al. (2019) [38]	SEA/Singapore/data from 2010 to 2015	Study found that 10 μg/m3 increase in particulate matter was associated with increases in nonaccidental (PM10 ER: 0.627%; 95% confidence interval (CI): 0.260–0.995% and PM2.5 ER: 0.660%; 95% CI: 0.204–1.118%) and cardiovascular mortality (PM10 ER: 0.897; 95% CI: 0.283–1.516 and PM2.5 ER: 0.883%; 95% CI: 0.121–1.621%). Acute effects were significant in the elderly (aged 65 and above) but not in non-elderly. Effects by other pollutants were minimal.
Ho, et al. (2018) [42]	SEA/Singapore/data from 2010 to 2015	There were 105,504 deaths during the study period. Moderate (Risk ratio/IRR = 1.05; 95% CI = 1.03–1.07) and unhealthy (Risk Ratio/IRR = 1.08; 95% CI = 1.03–1.14) PSI levels show significant association with all-cause mortality. Unhealthy temperatures (above 28.5 °C) also show significant association at a Risk Ratio/IRR = 1.04; 95% CI = 1.02–1.06. Each increment of 30 units in PSI on the same day and previous 1–5 days was significantly associated with 2.51–3.40% excess risk of mortality (*p* < 0.001). Each increment of 1 °C in temperature exhibited health implications in lag day 0–2 with 0.95%–2.00% excess risk of mortality (*p* < 0.05).
Tan, et al. (2019) [21]	SEA/Singapore/2015	Study participants’ median age was 30 years (IQR 26–34), and new psychosomatic symptoms were reported by 35 (47.3%). There was a modest but significant decrease in pulsatility index (PI) and resistivity index (RI) in the left MCA after haze exposure (PI: *p* = 0.026; RI: *p* = 0.021). Haze causes significant alterations in cerebral hemodynamics in susceptible individuals, probably responsible for various psychosomatic symptoms.
Ho, et al. (2019) [37]	SEA/Singapore/data from 2010 to 2015	Investigated association between air pollution and acute myocardial infarction (AMI) incidence in Singapore. Each 30-unit increase in PSI showed significant association with increased AMI occurrence with incidence risk ratio (IRR) of 1.04 and 95% confidence interval (95%CI) of 1.03–1.06. In the subgroup of ST-segment elevation myocardial infarction (STEMI), the IRR was 1.00 and 95%CI was 0.98–1.03; while among NSTEMI, the IRR was 1.08, and 95%CI was 1.05–1.10. Moderate and unhealthy PSI showed association with AMI occurrence with IRR 1.08 95%CI 1.05–1.11 and IRR 1.09 95%CI 1.01–1.18, respectively. Excess risk remained elevated through the day of exposure and for up to five day after exposure (>2 years for Ho et al.).

**Table 2:** Estimated incidence rate ratio of myocardial infarction for each 30-unit increment in pollutant standards index for the entire study cohort and by subgroups of demographic and clinical characteristics (*n* = 2191 Days). Table reproduced with explicit permission from the authors of [37].

Groups	Incidence Rate Ratio (95% CI)	*p*-Value
Entire cohort	1.04 (1.03–1.06)	0.001
Without overdispersion and autocorrelation	1.04 (1.03–1.06)	0.001
**Subgroups**		
**Age**		
65 y	1.04 (1.02–1.07)	0.001
65 y	1.05 (1.03–1.07)	0.001
**Sex**		
Male	1.06 (1.04–1.08)	0.001
Female	1.04 (1.01–1.06)	0.005
**Ethnicity**		
Chinese	1.05 (1.03–1.07)	0.001
Malay	1.05 (1.02–1.08)	0.002
Indian	1.04 (1.01–1.08)	0.014
**Subtype**		
STEMI	1.00 (0.98–1.03)	0.940
NSTEMI	1.08 (1.05–1.10)	0.001
**History of MI/CABG/PCI**		
Yes	1.05 (1.03–1.08)	0.001
No	1.05 (1.03–1.07)	0.001
**History of diabetes mellitus**		
Yes	1.06 (1.04–1.08)	0.001
No	1.05 (1.03–1.07)	0.001
**History of hypertension**		
Yes	1.05 (1.03–1.07)	0.001
No	1.06 (1.04–1.09)	0.001
**History of hyperlipidemia**		
Yes	1.05 (1.03–1.08)	0.001
No	1.05 (1.02–1.07)	0.001
**Current/former smoker**		
Yes	1.04 (1.02–1.07)	0.001
No	1.06 (1.04–1.08)	0.001
**Place of MI onset**		
Inpatient	1.13 (1.09–1.16)	0.001
Outpatient	1.02 (1.00–1.04)	0.029
